# Genetic and Virulence Profiles of Enteroaggregative *Escherichia coli* (EAEC) Isolated From Deployed Military Personnel (DMP) With Travelers' Diarrhea

**DOI:** 10.3389/fcimb.2020.00200

**Published:** 2020-05-20

**Authors:** Courtney D. Petro, Jeffrey K. Duncan, Yuliya I. Seldina, Anna Allué-Guardia, Mark Eppinger, Mark S. Riddle, David R. Tribble, Ryan C. Johnson, Clifton L. Dalgard, Gauthaman Sukumar, Patrick Connor, Nadia Boisen, Angela R. Melton-Celsa

**Affiliations:** ^1^Department of Microbiology and Immunolgy, Uniformed Services University of the Health Sciences, Bethesda, MD, United States; ^2^Walter Reed National Military Medical Center, Bethesda, MD, United States; ^3^Department of Biology, The University of Texas at San Antonio, San Antonio, TX, United States; ^4^South Texas Center for Emerging Infectious Diseases, San Antonio, TX, United States; ^5^Department of Preventative Medicine and Biostatistics, Uniformed Services University of the Health Sciences, Bethesda, MD, United States; ^6^Department of Anatomy, Physiology and Genetics, Uniformed Services University of the Health Sciences, Bethesda, MD, United States; ^7^The American Genome Center, Uniformed Services University of the Health Sciences, Bethesda, MD, United States; ^8^Collaborative Health Initiative Research Program, Henry Jackson Foundation, Bethesda, MD, United States; ^9^Military Enteric Disease Group, Academic Department of Military Medicine, Birmingham, United Kingdom; ^10^Department of Bacteria, Parasites and Fungi, Statens Serum Institut, Copenhagen, Denmark

**Keywords:** enteroaggregative, *Escherichia coli*, travelers' diarrhea, EAEC, mouse model, biofilm, *aap*, deployed military personnel

## Abstract

To discern if there was a particular genotype associated with clinical enteroaggregative *Escherichia coli* (EAEC) strains isolated from deployed military personnel (DMP) with travelers' diarrhea (TD), we characterized a collection of EAEC from DMP deployed to Afghanistan, Djibouti, Kenya, or Honduras. Although we did not identify a specific EAEC genotype associated with TD in DMP, we found that EAEC isolated at the first clinic visit were more likely to encode the dispersin gene *aap* than EAEC collected at follow-up visits. A majority of the EAEC isolates were typical EAEC that adhered to HEp-2 cells, formed biofilms, and harbored genes for aggregative adherence fimbriae (AAF), AggR, and serine protease autotransporters of *Enterobacteriaceae* (SPATEs). A separate subset of the EAEC had *aggR* and genes for SPATEs but encoded a gene highly homologous to that for CS22, a fimbriae more commonly found in enterotoxigenic *E. coli*. None of these CS22-encoding EAEC formed biofilms *in vitro* or adhered to HEp-2 cells. Whole genome sequence and single nucleotide polymorphism analyses demonstrated that most of the strains were genetically diverse, but that a few were closely related. Isolation of these related strains occurred within days to more than a year apart, a finding that suggests a persistent source and genomic stability. In an ampicillin-treated mouse model we found that an *agg4A*+ *aar*- isolate formed a biofilm in the intestine and caused reduced weight gain in mice, whereas a strain that did not form an *in vivo* biofilm caused no morbidity. Our diverse strain collection from DMP displays the heterogeneity of EAEC strains isolated from human patients, and our mouse model of infection indicated the genotype *agg4A*+ *aar*– and/or capacity to form biofilm *in vivo* may correlate to disease severity.

## Introduction

Enteroaggregative *Escherichia coli* (EAEC) is a causative agent of both acute and persistent diarrhea in adults and children (Okeke and Nataro, 2001). Clinical presentation of EAEC infection is characterized by watery unformed stool with low-grade fever (Okeke and Nataro, 2001). For civilian travelers, EAEC is typically the third most identified enteropathogen responsible for TD, whereas EAEC is the second most common bacterial etiologic agent of TD in military travelers (Zboromyrska et al., 2014; Zaidi and Wine, 2015; Porter et al., 2017; Olson et al., 2019). For those deployed military personnel (DMP), TD is a leading cause of lost duty-days (Porter et al., 2017).

EAEC was originally characterized by the capacity to adhere to epithelial cells in a “stacked-brick” pattern (Nataro et al., 1987). Additionally, EAEC display aggregative adherence (AA) to one another and to synthetic surfaces, forming robust biofilms on plastic and glass (Okeke and Nataro, 2001), as well as *in vivo* in the ampicillin (Amp)-treated mouse model (Zangari et al., 2013). There are five different types of AA fimbriae (AAF), designated AAF/I-V, that are specific to EAEC (Jonsson et al., 2015). The genes for the AAF are located on the plasmid of aggregative adherence (pAA), and except for one reported strain (Jonsson et al., 2017), EAEC only encode one of the five AAF types. In the prototype strain 042 (AAF/II), proper display of fimbriae on the bacterial surface is dependent on the dispersin protein, Aap (Sheikh et al., 2002). Secretion of dispersin occurs through a dedicated transport system encoded on the pAA (genes *aatPABCD*). All of the genes necessary for AA are encoded on the pAA plasmid. Additional genes on pAA that are important for adherence include *aggR*, which encodes a transcriptional regulator. AggR positively regulates expression of genes required for AA as well as other genes on both pAA and the chromosome (Morin et al., 2013). Two examples of genes commonly found in EAEC that are regulated by AggR include the type 6 secretion system (T6SS) effector of unknown function, *aaiC*, and *aatA*, which encodes a component of the dispersin transport system. Besides AggR, many EAEC encode Aar, a negative regulator of AggR (Santiago et al., 2014). Mutations that inactivate *aar* cause increased fimbrial expression and elevated AggR expression (Santiago et al., 2014). Finally, a regulator encoded by *eilA* was shown in some EAEC to influence the capacity to adhere to HEp-2 cells and form biofilms (Sheikh et al., 2006). Other factors that may contribute to adherence by EAEC include heat-resistant hemagglutinin (Bhargava et al., 2009), long polar fimbriae (Ross et al., 2015), *E. coli* common pilus (Avelino et al., 2010), and Pil (Dudley et al., 2006; Garcia et al., 2019).

Once EAEC have established adherence, they can further damage epithelium through the release of a variety of factors that include members of the family of serine protease autotransporters of the Enterobacteriaceae (SPATEs). EAEC may encode one or several class 1 or 2 SPATEs (see review Ruiz-Perez and Nataro, 2014). Class 1 SPATEs that may be found in EAEC include the plasmid-encoded toxin Pet, which causes loss of actin stress fibers in tissue culture cells and subsequent cell death (Canizalez-Roman and Navarro-Garcia, 2003), Sat, which may affect tight junctions (Guignot et al., 2007), and SigA, which contributes to cell death and fluid accumulation in a rat ileal loop model of *Shigella flexneri* (Al-Hasani et al., 2000). Class 2 SPATEs generally exhibit immunomodulatory activity. For example, Pic exhibits proteolytic, mucinolytic activities and contributes to serum resistance (Harrington et al., 2009). SepA is a class 2 SPATE also found in *S. flexneri* where it has been found to contribute to disruption of the epithelial layer in model systems (Benjelloun-Touimi et al., 1995; Maldonado-Contreras et al., 2017). Classification and regression tree analysis (CART) showed that *sepA* from EAEC was associated more frequently with diarrheal cases than with controls in a study looking at pediatric cases of infection in Mali (Boisen et al., 2012). CART analysis was also used to examine EAEC strains in a case-control study in Brazil. In that study, the combination of *pet* and *aafA* (encodes AAF/II) was correlated with disease (Lima et al., 2013). A later study found that strains that lacked *aar* and *pic* were associated with malnourished children in Brazil (Havt et al., 2017).

In this investigation, we characterized a collection of EAEC strains isolated from DMP enrolled in the Trial Evaluating Ambulatory Therapy of Travelers' Diarrhea (TrEAT TD) Study (Riddle et al., 2017) by whole genome sequencing and analysis, biofilm formation, adherence to HEp-2 cells, and, for a subset, the capacity to cause failure-to-thrive (FTT) in an Amp-treated mouse model with the goal of identifying a genotype more associated with TD in the DMP population.

## Materials and Methods

### Strain Characterization

A collection of 174 presumptive EAEC (positive by PCR for *aatA* and/or *aaiC*) isolated from 98 patients during the TrEAT TD study (Riddle et al., 2017) were sent to Uniformed Services University of the Health Sciences. A secondary PCR screen of the isolates in our laboratory found that some of the isolates did not have either *aatA* or *aaiC*, and those isolates were excluded from the study. For our purposes, we named the cultures with a single letter, E (Egypt), K (Kenya), or P (Peru) to indicate the referral lab location in which the bacteria were isolated, followed by a number, then V1 (initial clinic visit), V4 (day 7 after initial clinic visit), or V5 (day 21 after initial clinic visit) to indicate the clinic visit from which the cultures arose. Isolates that were positive in our laboratory by PCR for *aatA* and/or *aaiC* were then screened by PCR for AAF and SPATE genes as well as for *aggR* and *aar* and tested for resistance to Amp by overnight growth on Luria Bertani (LB) agar supplemented with 100 μg/ml Amp. For some patients, we received up to five isolates from the same visit. If those isolates had the same pattern of EAEC genes (*aatA, aaiC, aggR, aar*, AAF, and SPATEs) we moved forward with only one of the isolates.

### DNA Preparation and Sequencing

The presumptive EAEC strains were grown overnight on LB agar and a single colony was resuspended in 5 mls of LB and grown overnight at 37°C. Genomic DNA (gDNA) was prepared from overnight cultures with the DNeasy Blood and Tissue Kit (Qiagen). The gDNA concentration was quantified with the PicoGreen dsDNA Assay Kit (Thermo Fisher). Sequencing libraries were prepared with 1 ng gDNA input using the Nextera XT standard kit (Illumina FC-121-1024 and FC-121-1001) and the Nextera XT Index Kit v2 (96 indexes for 384 samples). Sequencing libraries were assessed for size distribution and absence of free adapter and adapter dimers using the AATI Fragment Analyzer and library concentration was determined using a quantitative PCR-based assay with the KAPA Library Quantification Kit on the Roche LightCycler 480 Instrument II. The libraries were normalized, pooled and sequenced on a NextSeq 500 (Illumina) using paired-end run conditions for 300 cycles (2x150 with dual index reads). Sequencing run data was demultiplexed using bcl2fastq Conversion Software 2.18 to generate FASTQ files.

### Generation of Genomic Contigs and Analysis

Illumina paired-end reads were assembled into larger contigs using the CLC Genomics Workbench platform following the standard procedure. Virulence genes and serotypes were identified from the genome assemblies through the Center for Genomic Epidemiology's Virulence Finder (Joensen et al., 2014), Serotype Finder (Joensen et al., 2015), ResFinder (acquired antibiotic resistance genes, Zankari et al., 2012), and the multi-locus sequence typing (MLST, Larsen et al., 2012) web interfaces, and the Virulence factor database (VFDB) web interface (Liu B. et al., 2019). Presence or absence of virulence genes was further confirmed using CLC Genomics Workbench. The absence of SPATE genes *boa* (GenBank #AY876285.1), *sha* (MH899684), *tagB* and *tagC* (GenBank # MH899681), and *tleA* (GenBank # KF494347) was confirmed with Clone Manager 9 software. Putative biofilm-associated genes *shf* (GenBank # AF134403.1), *fis* (GenBank # NC_000913.3), *yafK* (P0AA99.1), and *yfaL*/*ehaC* (GenBank # NZ_AFWC01000180.1) were detected with Clone Manager 9 software. Possible adherence genes for AIDA-I (*aida*, GenBank # JQ044409.1), CS22 (*cseA*, Genbank # AF145205.1 Pichel et al., 2000, the operons with *ecpA* (Genbank # FJ210911.1), type 1 fimbriae gene *fimA* (GenBank # X00981.1), long polar fimbriae operons 1 and 2 (detected by searching for *lpf* A 1 and 2 from prototype EAEC strain 55989, GenBank # NC_011748.1), and *pil* [GenBank accession AY686591.1 for pSerB1 (Dudley et al., 2006)], were detected by searching the assembled contigs with BLAST® or Clone Manager 9 software. The sequences from the EAEC from this study are available as part of Bioproject PRJNA576592 in the NCBI BioProject database (https://www.ncbi.nlm.nih.gov/bioproject/).

### Whole Genome Alignment (WGA) Phylogeny

Fifty of the EAEC genomes sequenced in this study along with the prototypical EAEC reference strain 042 genome (GenBank accession: NC_017626.1, Chaudhuri et al., 2010) were used to construct a whole genome-based phylogenetic tree. The WGS from E15V1A, K6V5, K32V4, K33V5, K29V1, and K36V1were not included in the analyses because we originally were unsure whether to include them because they lacked *aggR* and *aaiC* (E15V1A, K29V1, and K36V1—in which we subsequently found a variant *aaiC* gene), or because they lacked *aggR, aap* (we later identified an *aap*-like gene in these strains), and had only one SPATE. The phylogeny was inferred from whole genome alignments (WGA) using Mugsy 1.2.3 (Angiuoli and Salzberg, 2011) and RAxML 4.0 (Stamatakis, 2014) with 100 bootstrap replicates. The tree was visualized in Geneious 2019.1.2 (Kearse et al., 2012) and decorated with strain-associated metadata in Evolview v3 (Zhang et al., 2012; He et al., 2016; Subramanian et al., 2019).

### Core Genome SNP Phylogeny

To compute a SNP-based phylogeny for the sampled EAEC, we used a custom-built core genome (cg) SNP discovery pipeline described in more detail in Eppinger et al. (2010, 2011, 2014), Rusconi et al. (2016), and Hau et al. (2018), which is implemented on the open-source web-based bioinformatics platform Galaxy (Goecks et al., 2010). This high-resolution SNP discovery and typing strategy allowed us to put the sampled EAEC strains into a phylogenomic context and determine their individual phylogenetic relationships. We defined the chromosomal core genome as the set of genic and intragenic regions that do not contain phages, repeats, IS elements, plasmid regions, genomic islands, or other mobile genetic elements, which evolve at different rates and are not indicative of evolutionary relationships. These excluded regions were determined for the designated prototypical closed reference EAEC strain 042 (Chaudhuri et al., 2010) as follows: Repeats with NUCmer 3.22 (Delcher et al., 2003), prophages with PHASTER (Arndt et al., 2016, 2017), and IS elements with ISFinder (Siguier et al., 2006), ISEScan 1.7.1 (Xie and Tang, 2017), and ICEberg (Liu M. et al., 2019). The modular read-based SNP discovery pipeline contains the following workflow steps: (i) SNP discovery. Illumina reads of the 50 EAEC query strains were aligned with BWA-MEM (Li and Durbin, 2009) to the designated reference genome EAEC 042. The resulting alignments were processed with Freebayes 1.3.1 (Garrison and Marth, 2012) with the following threshold settings: mapping quality 30, base quality 30, coverage 10, and allelic frequency 0.75. The resulting SNP panel for each of the query genomes was used for further processing; (ii) SNP validation and filtering. We used several SNP curation strategies as described previously (Eppinger et al., 2010, 2011, 2014; Rusconi et al., 2016; Hau et al., 2018). Briefly, cataloged SNPs from each genome were merged into a single SNP panel and SNPs located within excluded regions were removed along with low quality alignments or misalignments, non-uniformly distributed regions and InDels, as previously described (Myers et al., 2009; Morelli et al., 2010; Eppinger et al., 2014). SNPs were further curated by extracting the surrounding nucleotides (40 nt) for each predicted SNP in the reference genome followed by BLASTn search against the query genomes (Altschul et al., 1990); (iii) SNP annotation and chromosomal distribution. Allelic status and chromosomal position of SNPs were recorded and for the biological relevance of the SNPs, polymorphisms were classified into intragenic or intergenic by mapping the SNPs to the 042 reference genome annotation (Manning et al., 2008; Bono, 2009; Chaudhuri et al., 2010; Leopold et al., 2010; Rusconi et al., 2016). In addition, we used a custom-developed genotyper tool to provide a summary of the SNP statistics and report on the number of individual genotypes in the phylogenetic network. (iv) SNP phylogeny. The curated panel of high quality SNPs served as the basis for phylogenetic reconstruction by maximum parsimony with PAUP v4.0a163 (Wilgenbusch and Swofford, 2003) with 100 bootstrap replicates. The majority rule consensus SNP tree was visualized in Geneious (R11.1.5) (Kearse et al., 2012) and decorated in Evolview (Zhang et al., 2012; He et al., 2016; Subramanian et al., 2019). Calculation of the consistency index in Mesquite (Maddison and Maddison, 2019) for each SNP allowed us to identify parsimony informative SNPs and flag homoplastic SNPs, as described previously (Eppinger et al., 2010, 2011, 2014; Rusconi et al., 2016; Hau et al., 2018).

### Biofilm Assay

Strains were grown overnight in LB broth with shaking. The following day, 10 μl of overnight culture was added to 1 ml Dulbecco's Modified Eagle Medium (DMEM, ThermoFisher Scientific, Waltham, MA) with 0.45% glucose and L-glutamine and 180 μl of the diluted sample was added in triplicate to a 96-well plate. The samples were incubated for 24 h at 37°C without shaking. After 24 h, the media was carefully aspirated and the biofilms washed once with phosphate-buffered saline (PBS). The biofilms were then fixed with 75% ethanol and allowed to dry completely. The biofilms were then stained with crystal violet, rinsed with water, and allowed to dry. The crystal violet was eluted with 100 μl of 75% ethanol and allowed to incubate for 5 min prior to reading absorbance at a wavelength of 592 nm.

### Adherence Assay

The adherence protocol was based on that of Cravioto et al. (1979). Briefly, HEp-2 cells at a concentration of 2.5 x 10^5^ cells/ml were added to an eight-well chamber slide and incubated overnight in DMEM with 10% fetal bovine serum and penicillin-streptomycin (Lonza) and gentamycin (Quality Biologicals). The same day, cultures of EAEC strains were inoculated into LB and incubated with shaking at 37°C for ~6 h. A 1:100 dilution of the culture was added to DMEM and the culture incubated overnight with shaking at 37°C. The following day, the HEp-2 cells were washed three times with PBS warmed to 37°C, then overlaid with the bacterial inoculum diluted into DMEM with 1% D-mannose for a final multiplicity of infection of 100. The infected HEp-2 cells were incubated for 3 h, then carefully washed three times with PBS, fixed with 0.8% glutaraldehyde/1%formaldehyde in PBS, washed, and stained with 20% Gibco Giemsa Stain solution (Fischer Scientific). Slides were imaged with an Olympus BX60 microscope. The adherence phenotypes were determined by two investigators blinded as to the strain observed according to the adherence descriptions by Nataro et al. (1987).

### Mouse Studies

Mouse studies were conducted in strict accordance with the recommendations of the *Guide for the Care and Use of Laboratory Animals*. All animal studies were approved by the Institutional Animal Care and Use Committee of the Uniformed Services University of the Health Sciences. Female C57BL/6 mice aged 5–6 weeks from Jackson Labs were used for all mouse experiments. Mice were fasted for ~16 h prior to bacterial inoculation and given drinking water supplemented with 2.5 g/l Amp (Corning). Mice were orally infected with 10^9^ to 10^11^ CFU in 100 μl by intragastric gavage. The inoculum was prepared from an overnight culture of EAEC grown in LB. The culture was pelleted by centrifugation, and the pellet resuspended in PBS at 1/10 of the original volume. Food was returned ~30 min post-infection (p.i.). Amp treatment continued throughout the course of the experiment. Fresh Amp water was prepared every 48 h. Mice were monitored for weight change for up to 42 days p.i. Weight change was determined as the weight p.i. compared to the weight of the mice the day before infection (d−1). In these studies, the FTT phenotype was assigned to mice that gained less weight compared to PBS mock-infected mice. Weight gain was compared by two way analysis of variance (ANOVA) with Dunnett correction for multiple comparisons. Colonization levels were determined through the course of the experiment, from fecal pellets homogenized in PBS (1:9 weight: volume), then serially diluted and plated onto LB agar plates supplemented with Amp (100 μg/ml).

### Preparation of Tissue Sections for Immunostaining

Formalin-fixed intestine was embedded in paraffin and then sectioned. Tissue sections were then placed on charged glass slides at HistoServ (Germantown, MD). Next, slides were deparaffinized in the Histoclear agent (National Diagnostics, Atlanta, GA) and rehydrated in a graded ethanol series. To increase antibody recognition of the antigen in tissue sections, slides were treated with antigen retrieval buffer [5 × AntigenPlus buffer, pH 10 (EMD Biosciences, San Diego, CA), diluted to 1 ×], heated in a microwave oven for 15 min at 50% power, cooled, and rinsed with water as done previously (Mohawk et al., 2010). Slides were then incubated overnight in blocking buffer (3% bovine serum albumin in PBS). To locate EAEC bound to or near the surface of intestinal tissue, slides with intestinal sections were immunostained with the appropriate anti-O serum (O92 for P73V1 and JM221, O99 for P433V1, Statens Serum Institute, Copenhagen Denmark). The anti-O serum was incubated with the tissue sections at a dilution of 1:50 in PBS for 1 h. The antiserum-exposed tissue sections were then washed, and secondary antibody conjugated to Alexa Fluor 488 was added for 1 h at a dilution of 1:200 in PBS. The slides were rinsed in PBS, 4′,6-diamidino-2-phenylindole (DAPI) Slowfade reagent (ThermoFisher Scientific, Waltham, MA) was added, and a coverslip was applied. Immunofluorescence of stained tissue sections was visualized with an Olympus BX60 microscope with a BX-FLA fluorescent attachment. Digital images of the fluorescent images were obtained with a SPOT RT charge-coupled-device digital camera (Diagnostic Instruments, Inc., Sterling Heights, MI).

## Results

### EAEC From DMP Were Heterogeneous in Virulence Factor Genes and Predicted Serotype Profiles

We isolated and sequenced gDNA from 56 presumptive EAEC isolates received from field laboratories in the TrEAT TD study. The sequences were assembled into contigs and then analyzed to determine the virulence gene profile and the predicted O and H antigen type. The operons for the known EAEC AAF variants I-V were identified in 35/56 of the strains in the collection (Table 1). The sequence for one strain (E11V1A) contained the genes for both *agg3A* (entire *agg3* operon was present) and *agg5A*, a phenomenon described previously (Jonsson et al., 2017), although in E11V1A there was only a single *agg3B* gene. Most of the isolates also had the operons for *E. coli* common pilus (Ecp), type I fimbriae, long polar fimbriae type 1 or 2, and/or Pil (Table 2). The gene for heat-resistant agglutinin 1, *hra1*, identified by Bhargava et al. (2009) as an accessory colonization factor, was found in six of the isolates with an AAF operon, and none of the isolates had the gene (*aida*) for AIDA-I production, an autotransporter reported to confer diffuse adherence in *E. coli* (Benz and Schmidt, 1989), Table 2. All of the EAEC with an AAF operon had *aap, aatA* [the entire operon (*aatPABCD*) was present in all strains that had *aatA*], and *aggR*, and, with the exception of the *agg4A*-positve strains, all had *aar* (Table 1). The gene that encodes *eilA* was only found in seven isolates. All of the AAF operon-positive isolates adhered to HEp-2 cells (Table 2).

**Table 1:** Virulence gene profile of EAEC with an aggregative adherence fimbrial gene isolated from DMP.

**Strain**	**AA fimbrial gene operon**					**Dispersin**		**Regulators**			** Type VI secretion system effector**	**SPATES/toxins**							**Predicted serotype**		** MLST^**#**^**
	***aggA***	***aafA***	***agg3A***	***agg4A***	***agg5A***	***aap***	***aatA^***wo***^***	***aar***	***aggR***	***eilA***	***aaiC^***wo***^***	***astA***	***pic***	***pet***	***sat***	***sepA***	***sigA***	***espI***	**O**	**H**	**ST**
E3V1C	+	−	−	−	−	+	+	+	+	−	+	−	+	−	+	−	−	−	NT	10	6259
E13V1E	+	−	−	−	−	+	+	+	+	−	+	−	+	−	−	+	+	−	181	4	678
E30V1A	+	−	−	−	−	+	+	+	+	−		−	−	−	−	−	−	−	21	27	295
E31V1B	+	−	−	−	−	+	+	+	+	−	+	−	+	−	+	−	−	−	NT	10	6259
K2V1	+	−	−	−	−	+	+	+	+	−	+	−	+	−	−	−	−	+	78	2	10
K11V5	+	−	−	−	−	+	+	+	+	−	+	+	+	−	+	+	−	+	93	8	5617
K22V1	+	−	−	−	−	+	+	+	+	−	+	−	+	−	−	−	−	+	78	2	10
K38V1	+	−	−	−	−	+	+	+	+	−	+	−	+	−	+	−	−	−	NT	10	6259
K39V1	+	−	−	−	−	+	+	+	+	−	+	−	+	−	+	−	−	−	NT	10	6259
P73V1	+	−	−	−	−	+	+	+	+	−	+	−	+	−	+	−	−	−	92	33	34
E13V1D	−	+	−	−	−	+	+	+	+	−	−	+	+	+	−	−	+	−	NT	7	2707
E14V1C	−	+	−	−	−	+	+	+	+	+	+	+	+	+	−	−	−	−	169	41	unk^∧^
E14V1D	−	+	−	−	−	+	+	+	+	+	+	+	+	+	−	−	−	−	44	18	414
E19V1A	−	+	−	−	−	+	+	+	+	+	+	+	+	+	−	−	−	−	44	18	414
E36V1A	−	+	−	−	−	+	+	+	+	+	+	+	+	+	−	−	−	−	44	18	414
K13V4	−	+	−	−	−	+	+	+	+	−	+	+	+	+	−	−	−	−	175	28	200
K24V1	−	+	−	−	−	+	+	+	+	−	+	+	+	+	−	−	−	−	175	28	200
K30V1	−	+	−	−	−	+	+	+	+	−	+	+	+	+	+	−	−	−	175	28	200
P109V1	−	+	−	−	−	+	+	+	+	−	+	+	+	+	−	−	−	−	175	27	200
P677V1	−	+	−	−	−	+	+	+	+	−	+	+	+	+	−	−	−	−	175	27	200
E11V1A	−	−	+	−	+	+	+	+	+	−	+^*^	+	+	+	−	−	−	+	NT	19	159
K4V4	−	−	+	−	−	+	+	+	+	−	+	−	+	−	−	−	−	+	21	2	10
K5V4	−	−	+	−	−	+	+	+	+	−	+	−	+	−	−	−	−	+	21	2	10
E8V1A	−	−	−	+	−	+	+	−	+	−	+	+	+	−	+	+	−	−	65	12	841
E16V1A	−	−	−	+	−	+	+	−	+	−	+	−	+	−	+	+	−	−	33	32	10
E17V1A	−	−	−	+	−	+	+	−	+	−	+	−	+	−	+	+	−	−	33	32	10
K21V5	−	−	−	+	−	+	+	−	+	−	−	−	−	−	−	−	+	−	59	19	1136
K26V1	−	−	−	+	−	+	+	−	+	−	+	−	+	−	+	+	−	−	33	19	10
K41V1	−	−	−	+	−	+	+	−	+	−	−	−	−	−	−	+	−	−	128ac	12	1326
P433V1	−	−	−	+	−	+	+	−	+	−	+	−	+	−	−	+	−	−	99	4	10
E10V5A	−	−	−	−	+	+	+	+	+	+	−	−	−	−	−	−	−	−	126	27	7425
E25V1B	−	−	−	−	+	+	+	+	+	−	+	−	+	−	−	−	−	−	99	10	34
P307V4	−	−	−	−	+	+	+	+	+	+	−	−	−	−	−	−	−	−	126	27	7425
P406V1B	−	−	−	−	+	+	+	+	+	+	+	−	+	−	−	−	−	−	126	27	unk
P415V1	−	−	−	−	+	+	+	+	+	−	+	+	+	−	+	−	−	+	3	2	10

Boxes colored orange indicate the genes originally used to screen for the EAEC.

Boxes colored green indicate presence of the gene.

Strain name boxes colored with purple indicate that the isolate was the sole pathogen identified in the stool sample.

# MLST-multi-locus sequence type.

^wo^whole operon present.

* The aaiC gene in this isolate is the same variant as found in C700-09 (Jonsson et al.).

∧ *unk-unknown MLST type*.

**Table 2:** Adherence gene or operon presence, and biofilm and adherence phenotype.

**Strain**	**AA-gene type operon**	***yfaL***	***hra1***	***ecpRABCDE***	**Type 1 fimbriae *fimBEAICDFGH***	***lpfA1B1C1D1E1***	***lpfA2B2C2D2***	***pilLMNOPQRSTUV***	**Biofilm^**∧**^**	**Adherence**
E3V1C	aggA	+	−	+	+	−	+	−	++	AA
E13V1E	aggA	+	−	+	−	+	+	+	+	AA
E30V1A	aggA	+	−	+	+	−	+	−	−	AA
E31V1B	aggA	+	−	+	+	−	+	−	+	AA
K2V1	aggA	+	−	+	+	−	−	−	+	AA
K11V5	aggA	+	−	+^*^	+	−	+	−	+	AA
K22V1	aggA	+	−	+	+	−	−	−	+	AA
K38V1	aggA	+	−	+	+	−	+	−	++	AA
K39V1	aggA	+	−	+	+	−	+	−	++	AA
P73V1	aggA	+	−	+	+	−	−	−	+	AA
E13V1D	aafA	+	−	+	+	+	+	+	−	AA
E14V1C	aafA	+	+	+	−	−	+	−	++	AA
E14V1D	aafA	+	+	+	−	−	+	−	+	AA/DA
E19V1A	aafA	+	+	+	−	−	+	−	++	AA
E36V1A	aafA	+	+	+	−	−	+	−	+	AA
K13V4	aafA	+	−	+	−	−	+	−	++	AA
K24V1	aafA	+	−	+	−	−	+	−	++	AA
K30V1	aafA	+	−	+	+	−	+	−	+	AA
P109V1	aafA	+	−	+	−	−	+	−	++	AA
P677V1	aafA	+	−	+	−	−	+	−	++	AA
E11V1A	agg3A/5A	+	−	−	+	−	−	−	+	AA
K4V4	agg3A	+		+	+	−	−	−	−	AA
K5V4	agg3A	+		+	+	−	−	−	−	AA
E8V1A	agg4A	+	+	+	+	−	+	−	+	AA
E16V1A	agg4A	+	−	+	+	−	−	−	+	AA
E17V1A	agg4A	+	−	+	+	−	−	−	+	AA
K21V5	agg4A	+	−	+	+	+	+	−	−	AA/LA
K26V1	agg4A	+	−	+	+	−	−	−	+	AA
K41V1	agg4A	+	−	+	+	−	+	−	+	DA/LA
P433V1	agg4A	+	+	^#^	+	−	−	−	+	AA
E10V5A	agg5A	+	−	+	+	−	−	−	+	AA
E25V1B	agg5A	+	−	+	+	−	−	+	+	AA
P307V4	agg5A	+	−	+	+	−	−	−	+	AA
P406V1B	agg5A	+	−	+	+	−	−	−	++	AA
P415V1	agg5A	+	−	+	−	−	−	−	+	AA

∧ Biofilm: biofilm values above the dotted line, + biofilm values between the dotted and dashed line, and, − biofilm values below the dashed line in Figure 2.

* Missing ecpR.

# Missing ecpR, ecpA and part of ecpB, but has ecpCDE.

*AA, aggregative adherence; DA, diffuse adherence; LA, localized adherence*.

All but three of the 35 AAF operon-positive isolates had genes for at least one SPATE (Table 1). Strain E30V1A that lacked any SPATE came from a patient who was also positive for enterotoxigenic *E. coli* (ETEC), so it is not clear whether the EAEC strain was responsible for the symptoms that patient exhibited. The other two isolates without a SPATE, E10V5A, and P307V4, were isolated during a follow-up visit, and no diarrheal symptoms were reported by the patients at that time.

An AAF operon was not identified for 21/56 (37.5%) presumptive EAEC isolates (Table 3). Similar rates for a lack of an AAF gene in EAEC were previously reported (Boisen et al., 2012; Jonsson et al., 2015). Nine of the 21 strains without a known EAEC AAF gene had a gene almost identical (97–99% homologous) to the gene (*cseA*) for colonization factor CS22 characterized in enterotoxigenic *E. coli* (ETEC) strains (Pichel et al., 2000). The presence of an operon for CS22 was recently identified in other EAEC (Boisen personal communication), and is also present in the *cseA*+ strains identified in this study (Supplemental Figure 1). All of the *cseA*-positive strains had *aggR* and at least one SPATE (Table 3), but lacked the genes for ETEC heat-stabile or heat labile toxin genes. However, we found that the isolates that have the CS22-like operon did not adhere to HEp-2 cells in our hands or showed minimal (a few bacteria could be found adherent to the HEp-2 cells) adherence (Table 4). ETEC with CS22 have been shown to adhere to Caco-2 cells (Pichel et al., 2000), but we did not test our strains on Caco-2 cells.

**Table 3:** Virulence gene profile of *E. coli* from DMP that lacked an AA fimbrial gene.

**Strain**	**CS22**	**Dispersin**		**Regulators**			**Type VI secretion system effector**	**SPATES/toxins**						**Predicted serotype**		**MLST**^**#**^
	***cseA**^***wo***^*	***aap***	***aatA**^***wo***^*	***aar***	***aggR***	***eilA***	***aaiC**^***wo***^*	***astA***	***pic***	***pet***	***sat***	***sepA***	***espI***	**O**	**H**	**ST**
E2V1A	+	+	+	+	+	−	+	+	+	−	−	+	−	39	49	2178
E3V1A	+	+	+	+	+	+	+	+	+	−	−	+	−	130	27	31
E3V1B	+	+	+	+	+	−	+	+	+	−	−	+	−	39	49	2178
E7V1A	+	+	+	+	+	−	+	+	+	−	−	+	−	39	49	2178
E9V1A	+	+	+	+	+	+	+	+	+	−	−	+	−	130	27	31
E11V5A	+	+	+	+	+	+	+	+	+	−	−	+	−	130	27	31
E32V5A	+	+	+	+	+	−	+	+	+	−	−	+	−	NT	10	5474
K18V1	+	+	+	+	+	−	+	+	+	−	−	+	−	9	21	155
K40V1	+	+	+	+	+	−	+	+	−	−	−	+	−	39	49	2178
K16V1	−	+	+	+	+	−	+	−	+	+	−	+	−	NT	19	278
K44V1	−	+	+	+	+	−	+	−	+	+	+	+	−	61	4	248
K45V1	−	+	+	+	+	−	+	−	+	+	+	+	−	61	4	248
E15V1A	−	+	+	−	−	−	−	−	−	−	−	−	−	NT	NT	206
K29V1	−	+	+	−	−	−	−	−	−	−	−	−	−	NT	27	10
K31V1	−	+	+	−	−	−	+	+	−	+	−	−	−	NT	5	6280
K36V1	−	+	+	−	−	−	+^*^	−	−	−	+	−	+	118/151	11	48
E18V1A	−			−	−	−	+	+	+	−	+	−	−	130	26	1178
E24V5C	−			−	−	−	+	+	+	−	+	−	−	130	26	1178
K6V5	−			−	−	−	+	+	+	−	−	−	−	99	33	10
K32V4	−			−	−	−	+	−	+	−	−	−	−	60	5	206
K33V5	−	−	−	−	−	−	+	−	+	−	−	−	−	60	5	206

Boxes colored orange indicate the genes originally used to screen for the EAEC.

Boxes colored green indicate the presence of the gene.

Strain name boxes colored in purple indicate that the isolate was the sole pathogen identified in the sample.

^wo^Whole operon present.

# MLST − multi-locus sequence type.

* The aaiC gene in this isolate is the same variant as found in C700-09 (Jonsson et al.).

Boxes colored blue indicate a gene with 47% homology to aap from 042.

*Boxes colored yellow indicate that a gene with low homology, 35%, was identified in the contigs for these strains*.

**Table 4:** Adherence genes or operons andbiofilm and adherence phenotype for strains without an AA-fimbrial gene operon.

**Strain**	***yfaL***	***CS22 operon^**#**^***	***hra1***	***ecpRABCDE***	***fimBEAICDFGH***	***lpfA1B1C1D1E1***	***lpfA2B2C2D2***	***pilLMNOPQRSTUV***	***Biofilm^**∧**^***	**Adherence**
E2V1A	+	+	−	+	−	−	+	+	−	not adherent
E3V1A	+	+	−	−	−	−	+	+	−	minimal
E3V1B	+	+	−	+	−	−	+	+	−	minmal
E7V1A	+	+	−	+	−	−	+	+	−	minimal
E9V1A	+	+	−	−	−	−	+	+	−	minimal
E11V5A	+	+	−	−	−	−	+	+	−	minimal
E32V5A	+	+	−	+	+	−	+	−	−	not adherent
K18V1	+	+	−	+	+	−	+	−	−	minimal
K40V1	+	+	−	+	−	−	+	−	−	minimal
K16V1	+	−	−	+	+	+	+	−	−	not adherent
K44V1	+	−	+	+	−	partial*	+	−	−	not adherent
K45V1	+	−	+	+	−	partial*	+	−	−	not adherent
E15V1A	+	−	−	+	+	−	−	−	−	minimal
K29V1	+	−	+	+	+	−	−	+	−	minimal
K31V1	+	−	−	−	+	−	−	+	−	minimal
K36V1	+	−	−	+	−	−	−	+	−	minimal
E18V1A	+	−	−	+	+	−	−	−	+	DA/LA
E24V5C	+	−	−	+	+	−	−	−	+	DA/LA
K6V5	+	−	−	+	+	−	−	−	+	LA/DA^~^
K32V4	+	−	−	+	−	−	−	−	−	DA
K33V5	+	−	−	+	−	−	−	−	−	minmial

# See Supplemental Figure 1 for operon structure.

∧ Biofilm: + biofilm values between the dotted and dashed line, and, − biofilm values below the dashed line in Figure 2.

^*^Missing lpfA1 and part of lpfB1.

~ This strain is higly adherent.

*DA, diffuse adherence; LA, localized adherence*.

There were 12 strains without an AAF gene or the CS22-like operon (Tables 3, 4). Of those isolates, K16V1, K44V1, and K45V1 had other adhesin gene markers, as well as *aap, attA, aar, aggR*, and three or more SPATEs, genotypes which marked them as EAEC, although they did not encode an AAF gene. We were unable to locate adhesin genes on the same contigs as genes that are typically found on the pAA plasmid (*aap, attA, aar, aggR, pet*, and *sepA*) in these latter strains. Of the final nine isolates listed in Table 3 without a known AAF gene or the CS22 operon, all lacked *aggR* and *aar* as well, but seven of those did have at least one SPATE. Eight of these nine isolates had the *ecp* operon that encodes the *E. coli* common pilus, an operon shown to be in other EAEC (Avelino et al., 2010). However, many of the strains that had the *ecp* operon did not adhere well to HEp-2 cells, which indicates that the presence of the *ecp* operon does not correlate with adherence. In contrast, two of the AAF gene-negative *ecp* operon-positive strains, E18V1A, and K6V5 did adhere well to HEp-2 cells (Table 4). The isolate (K31V1) that lacked an EAEC AAF gene, the CS22-like operon, *lpf* , or *ecp*, had the *pil* operon. In this collection, we found the *pil* operon in nine isolates without AAF operons and three of the 35 strains that had an EAEC AAF operon. However, the *pil*+ strains that were AAF gene-negative did not adhere well to HEp-2 cells in this study. Finally, none of the 21 isolates without an AAF operon had the gene (*aida)* for AIDA-I (associated with diffuse adherence).

All but 5/21 of the isolates without an AAF gene had the structural gene for dispersin, *aap*, and the dispersin transport system gene used for the original stool screening, *aatA* (Table 3), and in fact encode the entire operon for dispersin transport (*aatPABCD*). The five strains that lacked *aap* also lacked *aggR* and other genes that are markers for the pAA plasmid (Table 3). We suspected that one or more of these five strains may have lost the large plasmid during isolation, or carry a different plasmid, so we searched for other possible markers for the EAEC pAA. From that search, we found that 4/5 strains had a gene with 47% amino acid identity to *aap* (Supplemental Figure 2), and genes homologous to *aatPABCD*, with a lower overall identity that ranged from 30 to 47% depending on which gene in the operon was compared (not shown). The dispersin-like and dispersin-transport-like gene(s) were found in only one of the EAEC with an AAF gene, P406V1B. P406V1B also has the genes for the traditional dispersin and dispersin transport.

We next considered only those 23 EAEC isolates that came from visit 1 (sick visit) and which were the only identified pathogen from the stool (Table S1). The 23 isolates were 96% positive for *aap, aatA*, and *aaiC* and all had *yfaL*. The next most common genes were *pic* (87%), *aggR* (83%), and *aar* (70%). We also included the maximum number of stools in a 24 h period from the patients infected with the isolates listed in Table S1. The range for the number of stools from these isolates was 3–10, and the number from the isolates with *cseA* (4–7) was similar. In fact, no differences in the clinical picture from the patients with *cseA* isolates were noted overall.

To search for associations between EAEC genes present in any visit 1 (sick visit) isolate as compared to convalescent visits 4 and 5, we calculated the odds ratios (OR, Table 3). We found that the presence of *app* was associated with visit 1 (sick visit) as compared to visits 4 and 5 (follow-up visits), with an OR of 18.7 (*P* = 0.008), though the 95% confidence intervals were wide. None of the other EAEC genes correlated with visit 1 as compared to visits 4 and 5. We did a similar analysis for the EAEC that were identified as the only pathogen in the diarrheal stool compared to stool with EAEC and an additional pathogen(s), but found no correlations (Table 5).

**Table 5:** Odds ratios for the presence of EAEC genes in isolates from visit 1 compared to visits 4 and 5 and in isolates identified as the only pathogen compared to those which were not the only pathogen.

	**All strains**	**Visit 1**	Visits 4 and 5^*^	**OR (95% CI) visit 1 to visits 4 and 5**	**EAEC only**	**EAEC plus**	**OR (95% CI) EAEC only to EAEC plus**
N	56	43	13	N/A^**∧**^	23	33	N/A
AAF gene^=^	35	28	7	1.6 (0.5–5.7)	13	29	0.7 (0.2–2.0)
*aap^#^*	51	42	9	18.7^♦^(2.4–231.7)	22	29	3 (0.4–38.6)
*aar*	40	32	8	1.8 (0.5–6.7)	16	24	0.9 (0.3–2.7)
*aggR*	47	38	9	3.4 (0.9–15.7)	19	28	0.8 (0.2–3.1)
*aaiC*	48	38	10	2.3 (0.5–11.6)	22	26	5.9 (0.9–69.3)
*astA*	27	21	6	1.1 (0.3–3.7)	13	15	1.6 (0.6–4.7)
*pic*	46	36	10	1.5 (0.4–6.0)	20	26	1.8 (0.4–7.0)
*pet*	15	14	1	5.8 (0.8–66.1)	8	8	1.7 (0.6–5.0)
*sat*	17	15	2	2.9 (0.6–14.5)	9	8	2.0 (0.6–5.9)
*sepA*	20	17	3	2.2 (0.6–8.1)	9	11	1.3 (0.4–4.0)

* Visits 4 and 5 were 7 and 21 days after the first visit, respectively.

∧ N/A, not applicable.

= Any AAF gene.

# Does not include aap-like gene+ isolates.

♦ *P = 0.008*.

Over the entire collection of 56 isolates, there were a variety of O and H genotypes identified (Tables 1, 3). There were 21 predicted O antigen types with O130 and O175 present 5 times. The O antigen could not be typed in 11 strains. For the H antigen, the genes for H27, H6, and H2 were the most common. Some O:H combinations were more common than others: ONT:H10 (five strains), O39:H49 (four strains), O44:H18 (three strains), O126:H27 (three strains), O130:H27 (three strains), and O175:H28 (three strains). Notably, the serotypes for EAEC prototype strains JM221 (O92:H33) and 042 (O44:H18) were found in our collection. Finally, E14V1C was O169:H41, a serotype that has been linked to ETEC outbreaks in Asia and the U.S. (Beatty et al., 2004; Harada et al., 2013). Isolate E14V1C does have the heat stable enterotoxin (ST) gene (the only strain in the collection with that toxin gene) and may be a hybrid EAEC/ETEC strain. As expected for EAEC the strains had a variety of antibiotic resistance genes (Table S2).

We found that three patients appeared to be infected with two (E13V1D and E13V1E; E14V1C and E14V1D) or three (E3V1 A, B, and C) EAEC which differed by virulence gene make-up and/or serotype genes. This latter finding indicates that individuals may be infected with multiple EAEC at the same time, and demonstrates the importance of having multiple individual isolates from patients.

### Whole Genome Alignment and SNP Inferred Phylogenies of EAEC From DMP

**Figure 1:**
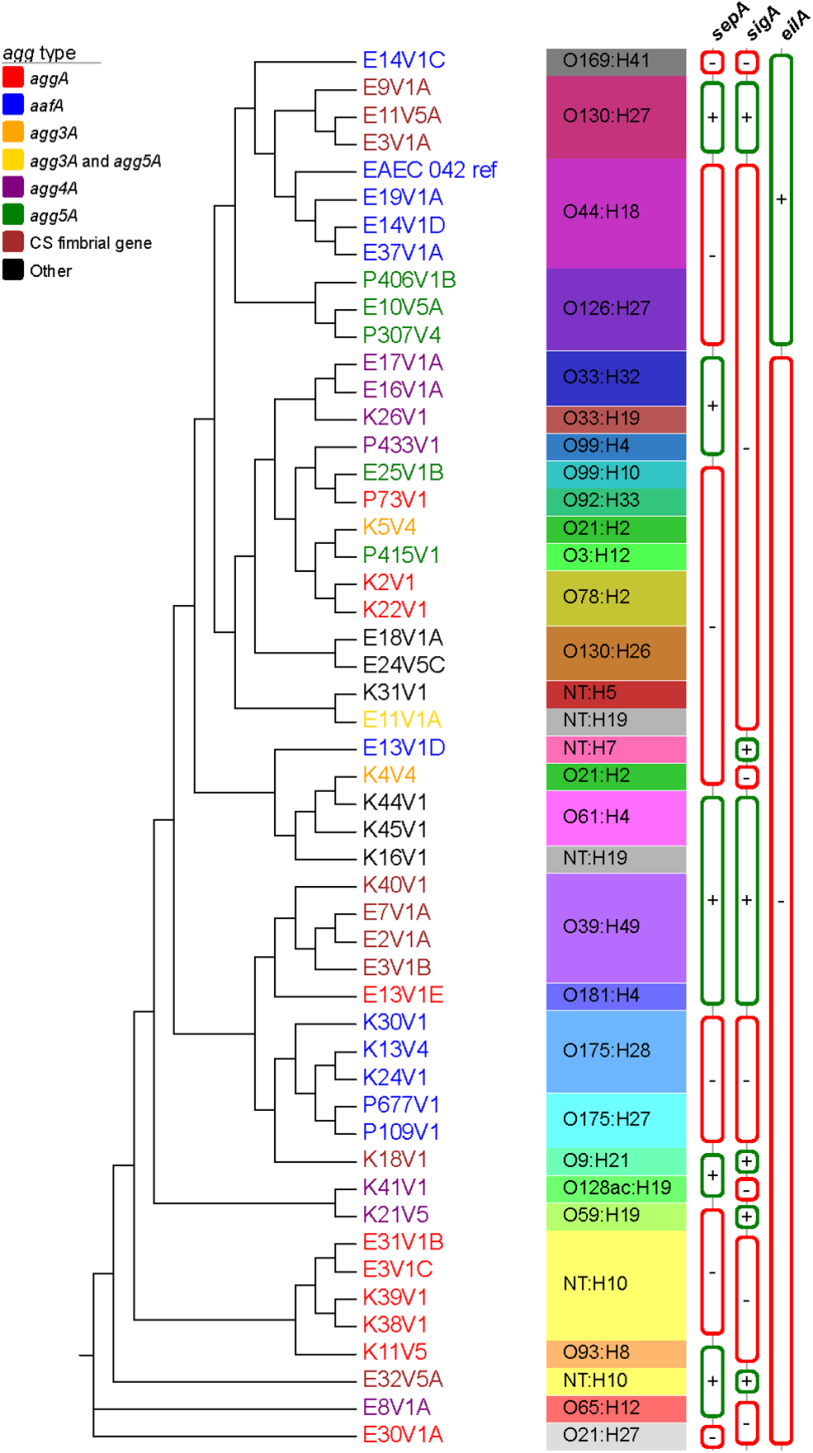
Whole genome phylogeny of sampled EAEC strains. The complete genomes of a total of 51 EAEC strains, which included the prototypical EAEC reference genome 042 (Chaudhuri et al., 2010), were aligned with Mugsy (Angiuoli and Salzberg, 2011). The phylogenetic tree was inferred using RAxML (Stamatakis, 2014) with 100 bootstrap replicates and decorated with the metadata in Evolview (Zhang et al., 2012; He et al., 2016; Subramanian et al., 2019). AAF type is depicted with different leaf colors, as shown in the legend. Serotype as well as *sepA, sigA*, and *eilA* status were also included in the tree. All nodes presented bootstrap values of >90. The tree topology partitions the isolates into distinct phylogenetic clusters in accordance with strain associated metadata.

A phylogenetic tree was established by aligning the whole genomes of 50 EAEC isolated from the DMP along with the genome of the prototype EAEC strain 042 (Figure 1). The WGA tree topology shows that the strains are separated into different genetic clusters and we observed correlations to AAF gene type, the predicted O and/or H antigen type, and somewhat by SPATE genes *sepA, sigA*, and the regulator *eilA*. The overall topology of the WGA phylogeny is largely mirrored by the SNP inferred phylogeny (Supplemental Figure 3). The SNP discovery yielded a total of 155,432 core genome SNPs (Table S3), of which 141,761 were parsimony informative. The latter is indicative of the diversity in the sampled EAEC strains, and is also reflected in the many branch points and individual clusters. However, in some cases EAEC from different patients in the same geographical area were found to be highly related and separated by fewer than 80 core genome SNPs from each other. Examples of such isolates include K38V1 and K39V1 (28 SNPs); E16V1A and E17V1A (55 SNPs); and E14V1D and E37V1A (6 SNPs) which were collected within the same day or week (see Supplemental Figure 3, black boxes). In contrast, other sets of genotypically highly similar isolates came from patients who reported illness 5 months [E3V1A and E11V5A (17 SNPs); K13V4 and K24V1 (34 SNPs)] or 1 year [E3V1C and E31V1B (79 SNPs)] apart (Supplemental Figure 3, green boxes). Further, there were two strains with just 46 SNPs from each other that were isolated from different DMP more than 1 year apart on different continents (E10V5A and P307V4, Supplemental Figure 3, red box). We also found examples of isolates that were more distantly related isolated from patients on the same day or 1 day apart [E7V1A compared to E8V1A (18,832 SNPs); E37V1A compared to the E13 strains (more than 55,600 SNPs); K40V1 compared to K38V1 and K39V1 (more than 19,100 SNPs)], findings that indicate that DMP may be exposed to multiple types of EAEC in the same geographic location.

### EAEC Strains Exhibit Diversity in the Capacity to Form a Biofilm and Adhere to Tissue Culture Cells *in vitro*

**Figure 2:**
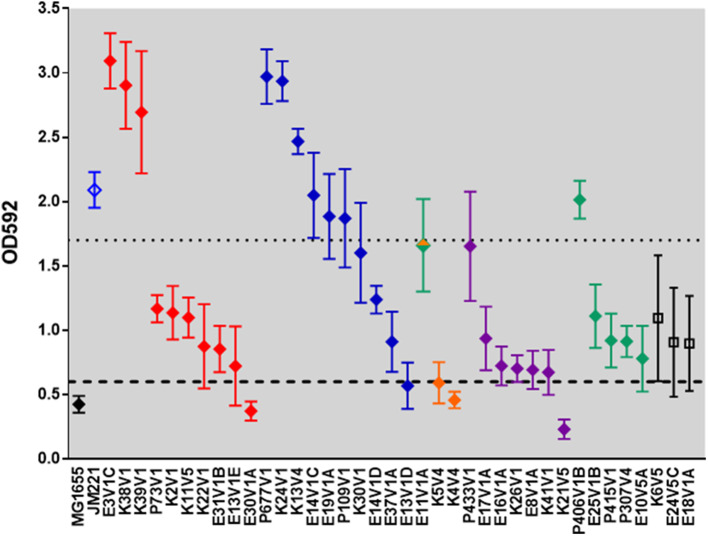
Biofilm-formation capacity of clinical isolates. The data represent the mean OD592 from at least three independent biofilm assays. The black diamond represents *E. coli* K12 strain MG1655. EAEC prototype strain JM221 is shown in blue. The filled diamonds are colored according to AAF operon type: *aggA* (red), *aafA* (blue), *agg3A* (orange), *agg4A* (purple), *agg5a* (green), and no AAF gene (black). Strain E11V1 is *agg3a*+ & *agg5a*+ and is shown with an orange and green diamond. Strains K6V5, E24V5C, and E18V1A did not have an AAF gene, and are indicated by open black squares. The dashed line is one standard deviation above the mean value for MG1655, and the dotted line is one standard deviation below the mean value for JM221. We classified high biofilm formers as those with values above the dotted line, and non-biofilm formers as those with values below the dashed line.

*In vitro* biofilm formation after 24 h was assessed for all EAEC strains (Figure 2, Tables 2, 4). In addition to the EAEC DMP strain collection, we used K12 strain MG1655 as a negative control, and prototype EAEC JM221 as a positive control. The biofilm-staining data in Figure 2 were grouped by fimbrial gene type and from the highest to lowest biofilm former to make the data easier to read. Within each fimbrial genotype we found variation in biofilm formation capacity, and a few strains did not form a biofilm by this assay (Figure 2). We found three strains (E18V1A, E24V5C, and K6V5) that lacked an AAF gene/operon were capable of moderate biofilm formation. The remainder of the strains without an AAF gene/operon were unable to form a biofilm (data not shown). All of the strains in this study had *yfaL*/*ehaC*, which if overexpressed in *E. coli* K-12 leads to enhanced biofilm formation (Roux et al., 2005), and *fis, yafK*, and *shf*, which if mutated, reduce biofilm formation (Sheikh et al., 2001; Fujiyama et al., 2008). However, all of the strains in this study were missing the start codon (ATG) of *shf* , so if the gene is expressed, the protein is likely truncated.

### Infection With EAEC Strain P433V1 Resulted in Reduced Weight Gain in Amp-Treated Mice

**Figure 3:**
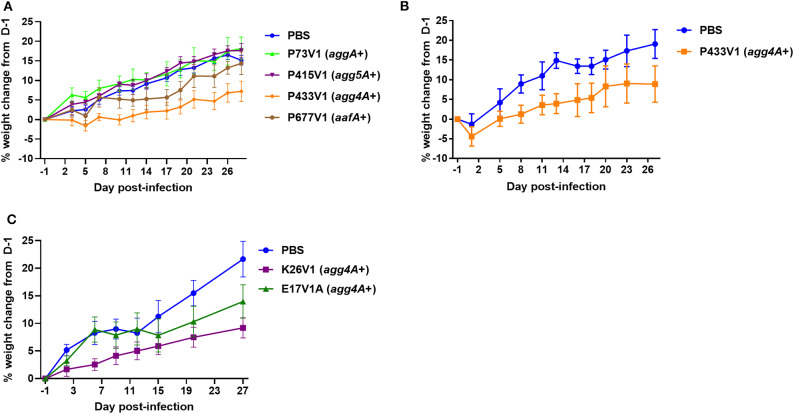
DMP strain P433V1 caused FTT in an Amp-treated adult-mouse model. **(A)** Mean weight change of mice infected with EAEC or mock-infected with PBS. Weights were compared with two-way ANOVA. Mice infected with P433V1 **(A)** showed differences in weight on day 10 and days 14–28 (*P* = 0.033 for days 10, 14, 28; 0.002 for days 17, 19, 21, and 26; and 0.0002 for day 24). There was no significant difference in weight gain between the other DMP strains (P73V1, P415V1, or P677V1) and the mock-infected mice. **(B)** Infection with strain P433V1 produced a reproducible FTT phenotype when compared to mock-infected mice. (*P* = 0.002 for day 20; 0.0001 for days 8, 11, and 18; <0.0001 for days 13, 16, 23, and 27 (*n* = 5 for mock-infected mice and *n* = 10 for P433V1). **(C)** Mean weight of mice (*n* = 5) infected with K26V1, E17V1A, or mock-infected with PBS. Mice infected with K26V1 showed differences in weight on days 20 and 27 (*P* = 0.015 for day 20 and 0.0001 for day 27). There was no significant difference in weight gain between E17V1A and mock-infected mice.

We used an Amp-treated mouse model to assess the pathogenesis of a subset of the EAEC collection. Because the Amp-treated mouse model was originally developed to characterize the O104:H4 2011 Shiga toxin-producing *E. coli* outbreak strain C227-11 (Zangari et al., 2013), and none of the EAEC strains in this collection had genes for Shiga toxin, we first tested the prototype EAEC strain JM221 in the model. We observed that mice infected with JM221 gained less weight over the course of the experiment compared to mock-infected controls, data not shown. We next tested a subset of EAEC from our DMP collection in the Amp-treated model. We began with Amp-resistant isolates from the Peru collection because those were the isolates we received first. Amp-treated mice infected with P73V1, P415V1, or P677V1 gained weight similar to that observed in the mock-infected controls (Figure 3A). However, mice infected with strain P433V1 (*agg4A*+ *aar*–) displayed significant FTT when compared to mock-infected controls (Figure 3A). We then tested P433V1 in a larger group of mice and again observed an FTT phenotype (Figure 3B). We also tested other *agg4A*+ and *aar*– strains in the mice. In a separate study, we found that strain K26V1, but not E17V1A caused a mild FTT phenotype (Figure 3C).

### Capacity to Form Biofilm *in vivo* Correlated With FTT Phenotype

**Figure 4:**
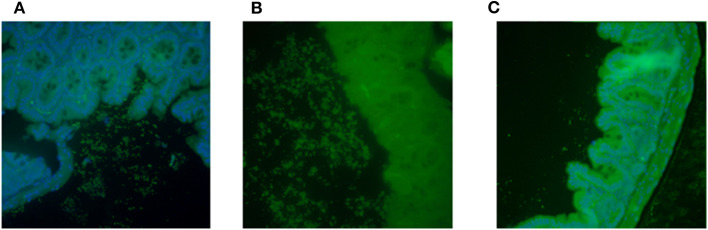
Capacity to form biofilm *in vivo* correlated with FTT phenotype. Intestines from EAEC-infected mice were stained with fluorescent antibody against the appropriate O-antigen. Mice infected with JM221 **(A)** or P433V1 **(B)** showed high levels of bacteria near the intestinal epithelium. Very few bacteria were observed in mice infected with P73V1 **(C)**. Sections were stained from three mice and representative images are shown.

While we were unable to identify an *in vitro* genotype unique for the isolates that caused FTT in the Amp-treated mice, we wanted to determine if the strains displayed differences in the capacity to form a biofilm *in vivo*. Intestinal sections from mice infected with JM221, P433V1, or P73V1 showed that there were high levels of bacteria in the sections from the mice that caused FTT (JM221 and P433V1), Figure 4, but that the sections from mice infected with P73V1 (did not cause FTT) had very few bacteria present. The colonization levels in the mice as determined by shedding into fecal samples was consistent throughout the course of the experiment among all strains at about 10^9^ CFU/g feces (not shown).

## Discussion

We set out to identify an EAEC genotype associated with TD in DMP. Although we did not identify a single such genotype, we did find a link between the presence of *aap* in the EAEC isolated at the acute clinic visit as compared to follow-up visits. Similarly, a study in Danish children found a link between *aap* and acute (as opposed to persistent) diarrhea in children (Hebbelstrup Jensen et al., 2017). The reduced number of EAEC with *aap* isolated from follow-up visits could indicate that strains without *aap* are less pathogenic than those with *aap*, and/or that the patients had mounted an immune response to dispersin. Therefore, it may be that dispersin would be a candidate vaccine antigen for EAEC. However, because we only had 12 isolates from follow-up visits in our study, the finding of strains that lack *aap* in patients without clinical disease needs to be confirmed. Finally, we found that some of the *agg4A* positive isolates caused FTT in an Amp-treated mouse model.

Our collection of EAEC isolates from DMP demonstrated genetic heterogeneity for the genes for AAF, other adhesins, and the SPATEs, with the exception of *pic* and gene *yfaL* which were found in >80% or all of the isolates, respectively. Most strains were also positive for *aggR, aar, aatPABCD, aap*, and *aaiA-Y*. One notable exception was that the *agg4A* strains lacked *aar*, a finding observed for other *agg4A*+ strains as well (Boisen, personal communication). For the SPATEs, the clearest associations were that the *aafA* strains had *pet* (a finding also noted by Lima et al., 2013), and all of the isolates with the CS22-like operon and most of the *agg4A* strains were *sepA* positive. The WGA and SNP analyses showed the genetic diversity among the EAEC, with many branch points and thousands of SNPs among the strains (see Table S1). However, the SNP analysis also demonstrated that some of the isolates were highly related with as few as 6 SNPs from each other in the core genome, suggesting a common source. These highly related strains came from patients who presented either within the same week or month, but a few were isolated more than a year apart. The finding of such genetically closely-linked isolates from patients who presented a year apart indicates that at least some of the strains are stable in that geographic region, perhaps re-circulating among the population. Furthermore, we ascertained that while most patients had only clonal isolates recovered, some patients were infected with multiple EAEC. These data collectively support the conclusion that many different EAEC are present in the same region. Another set of strains were just 46 SNPs apart in the core genome, but were isolated on different continents, a finding that suggests global distribution of EAEC.

We were somewhat surprised by the number of isolates that lacked an AAF gene that were associated with clinical disease and were the sole identified pathogen (Table 2, purple boxes and Table S1). This latter finding suggests that AAF-negative EAEC strains are associated with TD in this population of patients. It is also possible that some of the AAF-negative isolates did have a pAA plasmid at some point, but that it was lost during cultivation. However, the argument against that possibility is that many of these strains also had genes that are associated with pAA, such as *aggR, aap, pet*, and *sepA*. Therefore, it may be that the operon for CS22 replaced the AAF gene operon in these strains. Taken together these data suggest that the strains with the CS22-like fimbrial operon are EAEC that have acquired ETEC adherence genes. Finally, almost all of the isolates without an AAF gene had *aaiC*, a gene associated with EAEC.

We identified two strains that may have a novel adhesin mechanism as they lacked AAF genes, *hra1*, and *lpf* , and *pil* operons but adhered to HEp-2 cells. Although both strains did encode the *ecp* operon, and *yfaL*, the presence of *ecp* and *yfaL* did not correlate with adherence in our strains overall. These latter two isolates and two additional strains had a gene with high identity to *aap* (dispersin) and the operon for dispersin transport. The significance of the dispersin-like genes is not clear, but they could potentially contribute to adherence and/or biofilm formation in these strains.

Finally, we used a mouse model of infection established in our laboratory (Zangari et al., 2013; Boisen et al., 2019) to assess if a subset of the clinical isolates would be associated with morbidity in the animals. We found that Amp-treated mice infected with P433V1 or K26V1 showed FTT compared to mock-infected animals. Furthermore, P433V1 formed a strong biofilm *in vitro* and *in vivo*. Both P433V1 and K26V1 have *agg4A* and *sepA* but lack *aar*. A previous study showed an association between *sepA* and pediatric diarrhea cases compared to control patients (Boisen et al., 2012), and an *agg4A*+ Shiga toxin-producing *E. coli* (STEC)/EAEC hybrid strain that caused hemolytic uremic syndrome was described recently (Carbonari et al., 2019). However, the individual contributions of SepA and Agg4A in the disease process remain to be elucidated. In summary, we found that DMP with TD had EAEC that appeared to be similar to EAEC isolated from populations in other settings.

## Data Availability Statement

The datasets generated for this study can be found in the NCBI Bioproject, accession number PRJNA576592.

## Ethics Statement

The animal study was reviewed and approved by Uniformed Services University Institutional Animal Care and Use Committee.

## Author Contributions

CP, JD, YS, and AM-C conceived, planned, and carried the experiments with the support of all other authors. CP and AM-C wrote the manuscript with assistance from AA-G and ME. CD and GS provided experimental support and analysis, specifically with the sequencing. MR, DT, RJ, PC, and NB provided critical feedback and helped to shape the research and analysis.

## Conflict of Interest

The authors declare that the research was conducted in the absence of any commercial or financial relationships that could be construed as a potential conflict of interest.
