# Supplementary material for: Genetic and Virulence Profiles of Enteroaggregative Escherichia coli (EAEC) Isolated From Deployed Military Personnel (DMP) With Travelers' Diarrhea
Source: Front Cell Infect Microbiol. 2020 May 20;10:200. doi: 10.3389/fcimb.2020.00200 (PMC7251025; doi:10.3389/fcimb.2020.00200)
Supplement: Supplementary file 1 [file Data_Sheet_1.PDF]

Table S1 . Virulence gene profile of EAEC from visit 1 from DMP in which EAEC was the sole identified pathogen, and maximum number of stools in a 24 hour period from the patient with that isolate

Strain	AA fimbriae				CS22	dispersin		regulators		type VI secretion system effector	SPATES/toxins					predicted serotype		max stools
	aggA	*aafA*	*agg4A*	*agg5A*	*cseA*	*aap*	*aatA*	*aar*	*aggR*	*aaiC*	*astA*	*pic*	*pet*	*sat*	*sepA*	O	H	
E31V1B	+					+	+	+	+	+		+		+		NT	10	6
K2V1	+					+	+	+	+	+		+				78	2	10
K22V1	+					+	+	+	+	+		+				78	2	3
P73V1	+					+	+	+	+	+	+	+	+	+		92	33	4
E19V1A		+				+	+	+	+	+	+	+	+			44	18	5
K30V1		+				+	+	+	+	+	+	+	+	+		175	28	3
P109V1		+				+	+	+	+	+	+	+	+			175	27	8
P677V1		+				+	+	+	+	+	+	+	+			175	27	5
E8V1A			+			+	+		+	+	+	+		+	+	65	12	8
E16V1A			+			+	+		+	+		+		+	+	33	32	5
P433V1			+			+	+		+	+		+			+	99	4	7
P406V1B				+		+	+	+	+	+		+				126	27	8
P415V1				+		+	+	+	+	+	+	+		+		3	2	3
E2V1A					+	+	+	+	+	+	+	+			+	39	49	6
E7V1A					+	+	+	+	+	+	+	+			+	39	49	ND
E9V1A					+	+	+	+	+	+	+	+			+	130	27	7
K18V1					+	+	+	+	+	+	+	+			+	9	21	4
K16V1						+	+	+	+	+		+	+		+	NT	19	4
K44V1						+	+	+	+	+		+	+	+	+	61	4	ND
K29V1						+	+									NT	27	3
K31V1						+	+			+	+		+			NT	5	5
K36V1						+	+			+*				+		118/151	11	6
E18V1A										+	+	+		+		130	26	5

ND- no data available

Boxes colored orange indicate the genes originally used to screen for the EAEC.

*the *aaiC* gene in this isolate is the same variant as found in C700-09 (Jonsson et al.)

Box colored blue indicates a gene with 47% homology to *aap* from 042

Box colored yellow indicates a gne with low homology, 35%, was identified in the contig for this strain
