# Supplementary material for: Genetic and Virulence Profiles of Enteroaggregative Escherichia coli (EAEC) Isolated From Deployed Military Personnel (DMP) With Travelers' Diarrhea
Source: Front Cell Infect Microbiol. 2020 May 20;10:200. doi: 10.3389/fcimb.2020.00200 (PMC7251025; doi:10.3389/fcimb.2020.00200)
Supplement: Supplementary file 2 [file Data_Sheet_2.PDF]

Table S2 Antibiotic resistance genes present in the EAEC from DMP

	Trimethoprim	Aminoglycoside	Beta-lactam	Phenicol	Sulphonamide	Tetracycline	Macrolide
**E3V1B**			*blaCTX-M-15*		*sul1*		
**E2V1**			*blaCTX-M-15*		*sul1*		
**E3V1A**	*dfrA1*	*aadA1*	*blaTEM-1b*	*catA1*	*sul1*	*tet(A)*	*mdf(A)*
**E3V1C**							*mdf(A)*
**E7V1**			*blaCTX-M-15*		*sul1*		
**E8V1**		*aadA5*	*blaCTX-M-15, blaTEM-1B*		*sul1*	*tet(B)*	*mdf(A), mph(A)*
**E9V1**	*dfrA1*	*aadA1*	*blaTEM-1B*	*catA1*	*sul1*	*tet(A)*	*mdf(A)*
**E10V5A**	*dfrA1*	*aadA1, aph(3')-lb, aph(6)-ld*	*blaTEM-1B*			*tet(B)*	*mdf(A)*
**E11V1**	*dfrA7*	*aph(3')-lb, aph(6)-ld*	*blaTEM-1B*		*sul2*	*tet(A)*	*mdf(A)*
**E11V5**	*dfrA1*	*aadA1*	*blaTEM-1B*	*catA1*	*sul1*	*tet(A)*	*mdf(A)*
**E13V1D**	*dfrA8*	*aph(3')-lb, aph(6)-ld*	*blaTEM-1B*		*sul2*	*tet(B)*	*mdf(A)*
**E13V1E**	*dfrA7*	*aph(3')-lb, aph(6)-ld*	*blaTEM-1B*		*sul2*		*mdf(A)*
**E14V1C**	*dfrA7*	*aph(3')-lb, aph(6)-ld*	*blaTEM-1B*	*catA1*	*sul2*	*tet(B)*	*mdf(A)*
**E14V1D**	*dfrA7*	*aph(3)'lb, aph(6)-ld*	*blaTEM-1B*	*catA1*	*sul2*	*tet(A)*	*mdf(A)*
**E16V1A**	*dfrA7*	*aph(3)'lb, aph(6)-ld*	*blaTEM-1B*		*sul2*	*tet(A)*	*mdf(A)*
**E17V1**	*dfrA7*	*aph(3)'lb, aph(6)-ld*	*blaTEM-1B*		*sul2*	*tet(A)*	*mdf(A)*
**E18V1**						*tet(B)*	*mdf(A)*
**E19V1**		*aph(3')-lb, aph(6)-ld*		*catA1*	*sul2*	*tet(A)*	*mdf(A)*
**E24V5C**						*tet(B)*	*mdf(A)*
**E25V1B**	*dfrA1*	*aadA1, aph(3')-lb, aph(6)-ld*	*blaTEM-1B*	*catA1*	*sul2*		*mdf(A)*
**E30V1**			*blaTEM-1B*			*tet(B)*	*mdf(A)*
**E31V1B**	*dfrA1*	*aadA1, aph(3')-lb, aph(6)-ld*			*sul2*		*mdf(A)*
**E32V5**	*dfrA1*	*aph(6)-ld*	*blaTEM-1B*		*sul2*	*tet(B)*	*mdf(A)*
**E37V1**	*dfrA7*	*aph(3')-lb, aph(6)-ld*	*blaTEM-1B*	*catA1*	*sul2*	*tet(A)*	*mdf(A)*
**K2V1**	*dfrA7*	*aph(3')-lb, aph(6)-ld*	*blaTEM-1B*		*sul1, sul2*	*tet(A)*	*mdf(A)*
**K4V4**	*dfrA7*	*aph(3')-lb, aph(6)-ld*	*blaTEM-1B*		*sul2*		*mdf(A), mph(A)*
**K5V4**	*DfrA14*	*aph(3')-lb, aph(6)-ld*	*blaTEM-1B*		*sul2*		*mdf(A), mph(A)*
**K11V5**							*mdf(A)*
**K13V4**	*dfrA7*	*aph(3')-lb, aph(6)ld*	*blaTEM-1B*	*catA1*	*sul2*		*mdf(A)*
**K18V1**	*dfrA14, dfrA7*	*aph(3')-lb, aph(6)ld*	*blaTEM-1B*		*sul2*	*tet(A)*	*mdf(A)*
**K21V5**							*mdf(A)*
**K22V1**	*dfrA7*	*aph(3')-lb*	*blaTEM-1B*		*sul1, sul2*	*tet(A)*	*mfd(A)*
**K24V1**	*dfrA7*	*aph(3')-lb, aph(6)-ld*	*blaTEM-1B*	*catA1*	*sul2*		*mdf(A)*
**K26V1**	*dfrA8*	*aph(3')-lb, aph(6)-ld*	*blaTEM-1B*		*sul2*	*tet(B)*	*mdf(A)*
**K30V1**	*dfrA5*	*aph(3')-lb, aph(6)-ld*			*sul1,sul2*	*tet(A)*	*mdf(A)*
**K38V1**						*tet(A)*	*mdf(A), mph(A)*
**K39V1**						*tet(A)*	*mdf(A), mph(A)*
**K40V1**	*dfrA17*	*aadA5, aph(3')-lb, aph(6)-ld*	*blaTEM-1B*	*catA1*	*sul1, sul2*		*mdf(A), mph(A)*
**K41V1**	*dfrA7*	*aph(3')-lb, aph(6)-ld*	*blaTEM-1B*		*sul, sul2*	*tet(A)*	*mdf(A)*
**K16V1**	*dfrA7*	*aph(3')-lb, aph(6)-ld*	*blaTEM-1B*		*sul2*		*mdf(A)*
**K31V1**	*dfrA14*	*aph(6)-ld*			*sul2*	*tet(A)*	*mdf(A)*
**K44V1**	*dfrA8*	*aph(3')-lb, aph(6)-ld*	*blaTEM-1C*		*sul2*	*tet(A)*	*mdf(A)*
**K45V1**	*dfrA8*	*aph(3')-lb, aph(6)-ld*	*blaTEM-1C, blaTEM-1B*		*sul2*	*tet(A)*	*mdf(A)*
**P73V1**	*dfrA8*	*StrA, strB*	*blaTEM-1B*		*sul2*	*tet(B)*	*mdf(A)*
**P109V1**							*mdf(A)*
**P307V4**	*dfrA1*	*aadA1, strA, strB*			*sul2*	*tet(B)*	*mdf(A)*
**P406V1B**	*dfrA1*	*strA, strB, aadA1*			*sul2*	*tet(B)*	*mdf(A)*
**P415V1**	*dfrA7*	*strA, strB*	*blaTEM-1B*	*catA1*	*sul1, sul2*	*tet(A)*	*mdf(A)*
**P433V1**	*dfrA7*	*strA, strB*	*blaTEM-1B*		*sul1, sul2*	*tet(A)*	*mdf(A)*
**P677V1**	*dfrA8*	*strA, strB*	*blaTEM-1B*		*sul2*		*mdf(A)*
