# Supplementary material for: Genetic and Virulence Profiles of Enteroaggregative Escherichia coli (EAEC) Isolated From Deployed Military Personnel (DMP) With Travelers' Diarrhea
Source: Front Cell Infect Microbiol. 2020 May 20;10:200. doi: 10.3389/fcimb.2020.00200 (PMC7251025; doi:10.3389/fcimb.2020.00200)
Supplement: Supplementary file 4 [file Data_Sheet_4.PDF]

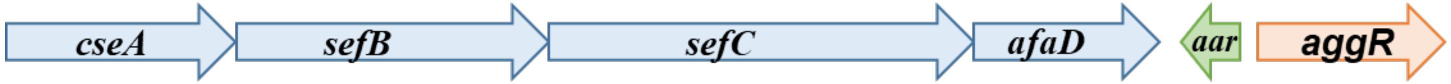

Supplemental Fig 1. Nine of the EAEC isolates had the gene for CS22, *cseA*, along with *sefB*- *sefC*- and *afaD*-like genes. Eight of the nine had *aar* and *aggR* genes downstream of the *afaD*-like gene on the same contig (E32V5A was the exception). The predicted amino acid sequence for CS22 from the nine EAEC isolates had 3 or 4 amino acid changes compared to the published CS22 sequence. Not to scale

```
042        mkkikfvifsgilgislnafaggsgwnadnvdpsqcikqsgvqyt------ynsgvsvcmqglnegkvrgvsvsgvfyyndgttsnfkgvvtpstpvntnqdinktnkvgvqkyraltewvk*
P433V1     mkkikfvifsgilgislnafaggsgwnadnvdpsqcikqsgvqyt------ynsgvsvcmqglnegkvrgvsvsgvfyyndgttsnfkgvvtpstpvntnqdinktnkvgvqkyraltewvk*
E18V1A     mkky---ilvalaglslnafaggsgwnpdevdpsrcvmamgagstsagyigyrqdnsecmqginegkvkgvnveghhefkdgtrdtfsgfvsptsplvlktdiskvnkvgiknwsykgtwvk*
E24V5C     mkky---ilvalaglslnafaggsgwnpdevdpsrcvmamgagstsagyigyrqdnsecmqginegkvkgvnveghhefkdgtrdtfsgfvsptsplvlktdiskvnkvgiknwsykgtwvk*
K6V5       mkky---ilvalaglslnafaggsgwnpdevdpsrcvmamgagstsagyigyrqdnsecmqginegkvkgvnveghhefkdgtrdtfsgfvsptsplvlktdiskvnkvgiknwsykgtwvk*
K32V4      mkky---ilvalaglslnafaggsgwnpdevdpsrcvmamgagstsagyigyrqdnsecmqginegkvkgvnveghhefkdgtrdtfsgfvsptsplvlktdiskvnkvgiknwsykgtwvk*
```

Supplemental Fig. 2. Alignment of *aap* (042 and P433V1) and *aap*-like genes from P433V1, E18V1A, E24V5C, K6V5, and K32V4.

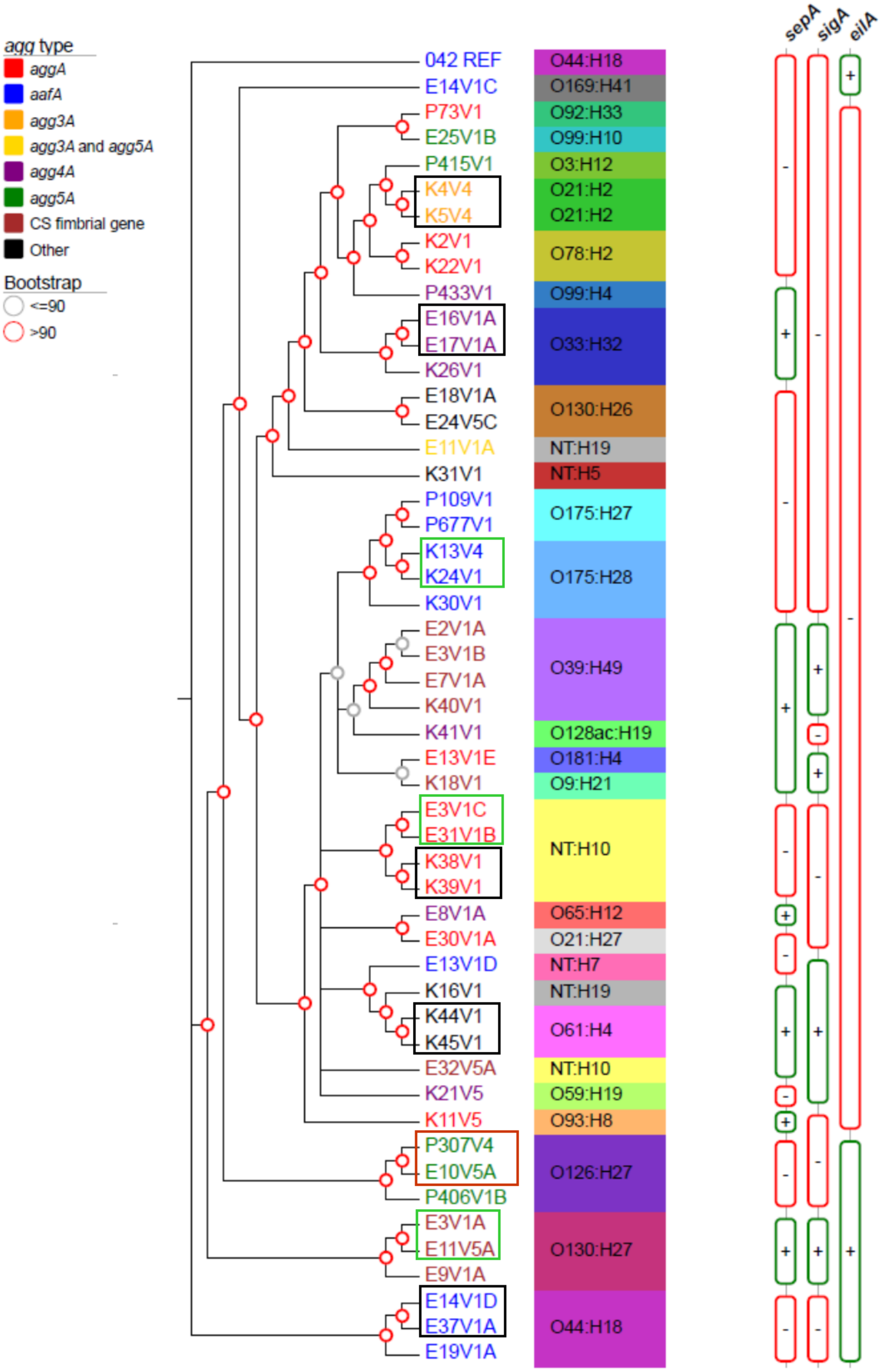

Supplemental Fig. 3. SNP phylogeny of samples EAEC strains. Comparison of 51 sequenced EAEC genomes yielded a total of 155,432 SNPs (Supplemental Table S1), of which 141,761 were parsimony informative. The tree shown is a majority-consensus tree of 126 equally parsimonious trees. Trees were recovered using a

heuristic search in PAUP (1) with 100 bootstrap replicates. Bootstrap values >90 are shown as red circles in the tree nodes. Isolates boxed in black were collected within the same day or week, in green were isolated 5 months or more apart, and in red were isolated a year apart (on different continents). The core genome SNP values that separate the boxed strains are as follows along with the clinic visit date difference: [K4V4 & K5V4 (21 SNPs, 4 days apart)]; [E3V1A & E11V5A (17 SNPs, 6 months apart)]; [E16V1A & E17V1A (55 SNPs, 5 days apart)]; [K13V4 & K24V1 (34 SNPs, 5.5 months apart)]; [E3V1C & E31V1B (79 SNPs, 1 year apart)]; [K38V1 & K39V1 (28 SNPs, same day)]; [K44V1 & K45V1 (20 SNPs, 2 days apart)]; [P307V4 & E10V5A (46 SNPs, 18 months apart)]; and [E14V1D & E37V1A (6 SNPs, 3 days apart)]. A comparison of the topologies of the SNP and WGA (**Fig. 1**) trees indicates that the major clusters are maintained.

Reference

1. **Wilgenbusch JC, Swofford D**. 2003. Inferring evolutionary trees with PAUP*. Curr Protoc Bioinformatics Chapter 6:Unit 6 418428704
